# Anti-neuraminidase and anti-hemagglutinin immune serum can confer inter-lineage cross protection against recent influenza B

**DOI:** 10.1371/journal.pone.0280825

**Published:** 2023-01-23

**Authors:** João Paulo Portela Catani, Tine Ysenbaert, Anouk Smet, Marnik Vuylsteke, Thorsten U. Vogel, Xavier Saelens

**Affiliations:** 1 VIB-UGent Center for Medical Biotechnology, VIB, Ghent, Belgium; 2 Department of Biochemistry and Microbiology, Ghent University, Ghent, Belgium; 3 Gnomixx, Melle, Belgium; 4 Sanofi, Research North America, Cambridge, Massachusetts, United States of America; University of South Dakota, UNITED STATES

## Abstract

Influenza B viruses (IBV) are responsible for a considerable part of the burden caused by influenza virus infections. Since their emergence in the 1980s, the Yamagata and Victoria antigenic lineages of influenza B circulate in alternate patterns across the globe. Furthermore, their evolutionary divergence and the appearance of new IBV subclades complicates the prediction of future influenza vaccines compositions. It has been proposed that the addition of the neuraminidase (NA) antigen could potentially induce a broader protection and compensate for hemagglutinin (HA) mismatches in the current vaccines. Here we show that anti-NA and -HA sera against both Victoria and Yamagata lineages have limited inter-lineage cross-reactivity. When transferred to mice prior to infection with a panel of IBVs, anti-NA sera were as potent as anti-HA sera in conferring protection against homologous challenge and, in some cases, conferred superior protection against challenge with heterologous IBV strains.

## Introduction

Influenza A and B viruses co-circulate and cause infections in human with clinical manifestation that are indistinguishable, with similar rates of hospitalization and death in the overall population [1, 2]. In contrast to influenza A the influenza B virus (IBV) does not have an established animal reservoir, limiting its pandemic potential. Although IBV causes nearly 25% of influenza virus infections [3], in children, influenza B is associated with more severe disease and increased influenza-associated deaths than influenza A [4, 5]. Evidence indicates that flu also causes additional burden with health impacts including cardiovascular events and the exacerbation of chronic underlying conditions. Vaccination is the most effective strategy to protect against seasonal influenza infection and protecting beyond flu meaning preventing further complications [6].

The IBV was first isolated in the 1940s and antigenic and genetic studies in the early 1990s showed that IBV has diverged in the 1970s into 2 distinct antigenic lineages: the Yamagata and Victoria lineages, which have circulated globally since 1985 [7–9]. These 2 lineages exhibit an alternating pattern of predominance among influenza seasons, likely reflecting the population immunity [9].

The inability to accurately predict the predominant IBV lineage led to the development of quadrivalent influenza vaccines that include a Yamagata (Yam) and a Victoria (Vic) IBV strain next to the two influenza A virus strains [10].

The evolution of IBV is shaped by a set of interactions including the epidemiological dynamics of IBV and IAV [11]. The evolutionary dynamics of IBV strains are characterized by the accumulation of amino acid substitution in the hemagglutinin (HA) and neuraminidase (NA) and by intra- and inter-lineage gene reassortments [11]. HA and NA are the major glycoproteins on the IBV surface with HA being the main or, in case of Flublok, the sole antigen component in currently licensed influenza vaccines. HA and NA gene segments have the highest ratio of non-synonymous over synonymous mutations, indicating strong immune pressure on those proteins [11, 12]. Amino acid substitutions and deletions are mostly located on the antigenic head regions of HA and NA and constitute the mechanisms of the most recent adaptations [12]. Interestingly, early in 2000s, the NA segment of the Victoria lineage was replaced by the one of the Yamagata lineage [13–15]. However, surveillance data from 2008 onwards indicate very limited inter-lineage reassortments, suggesting that IBVs with fit genomes have evolved in the human population, which now outcompete the emergence of new reassortants [12].

The induction of an anti-HA response is considered the gold standard for influenza vaccine potency. Therefore, HA antigenicity and breadth of protection are well understood. The antigenicity of NA, however, has been poorly studied and an even more limited number of studies aimed to determine the protective potential of IBV NA as immunogen [16, 17]. Still, broad cross-reactive anti-NA antibodies have been described and were shown to be protective in mouse models [18–20]. Despite NA’s protective potential, the induction of an anti-NA immune response is limited or absent in current vaccines [21–23]. Supplementation of NA, on the other hand, has been proposed to increase influenza vaccine effectiveness [24].

To fully evaluate the potential of NA as a protective vaccine antigen against IBV, understanding its antigenicity is crucial. Considering the relatively recent replacement of Victoria NA by Yamagata NA, it could be expected that a single NA antigen could confer cross-reactive antibodies against both circulating lineages. To address this, we generated a panel of anti-IBV HA and anti-IBV NA sera in mice and evaluated their cross-reactivity, cross-inhibition and protection against a panel of Yamagata and Victoria lineage IBV strains that correspond to the vaccine strains that were recommended from 2007 until 2020.

## Results

### Anti-NA and HA antibodies have limited cross-lineage reactivity

To generate immune sera, BALB/c mice were primed and boosted with 1 μg of AF03 adjuvanted recombinant tetrabrachion-stabilized soluble tetrameric NA (tetNA) or recombinant HA (rHA) derived from B/Brisbane/60/2008 (Bris), B/Colorado06/2017-like (Col) or B/Phuket/3073/2013 (Phu). Two weeks after the second immunization, the mice were terminally bled and the obtained sera pooled.

**Fig 1:**
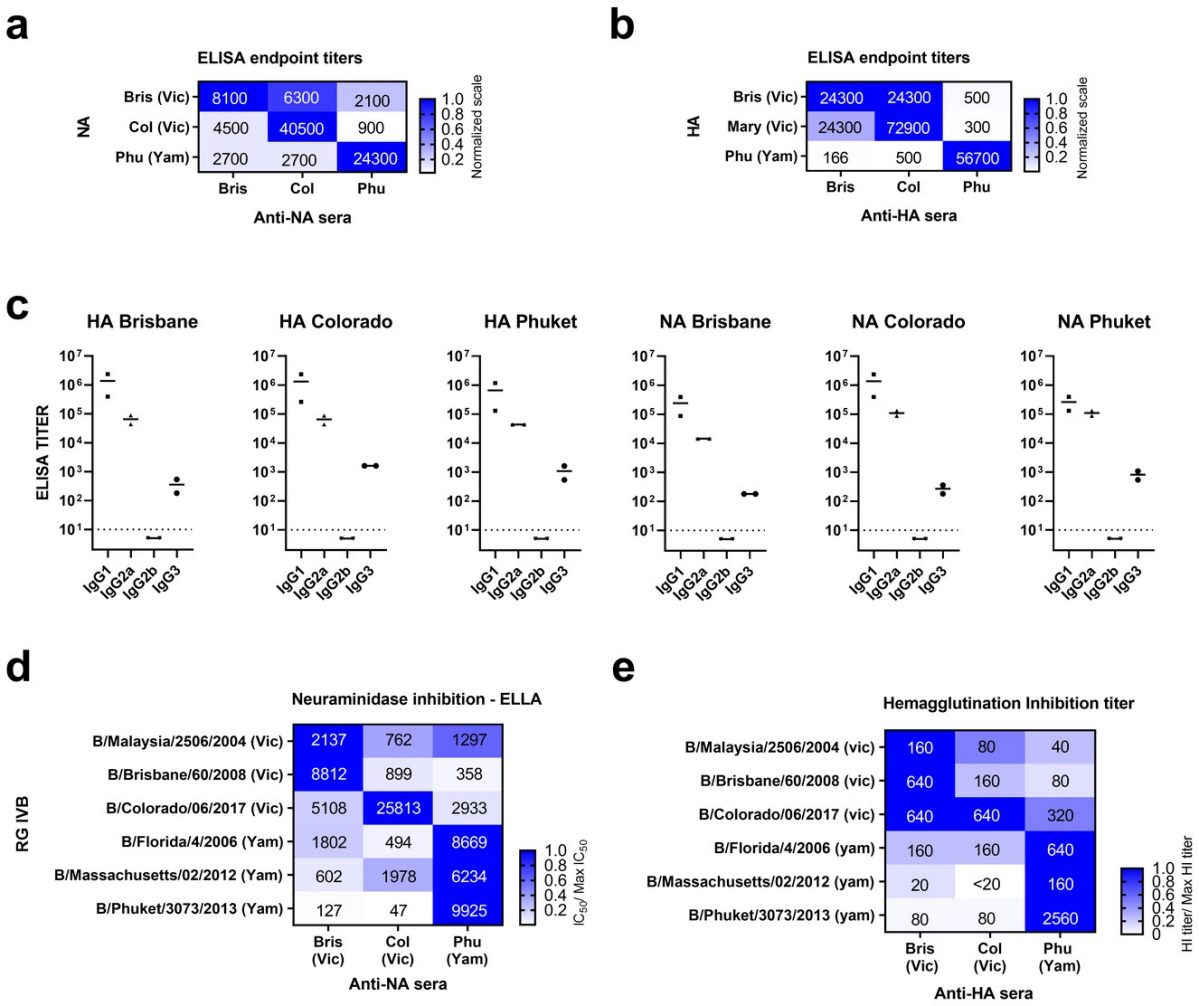
Characterization of anti-NA and anti-HA immune sera. (a) tetNA-specific endpoint IgG titers were determined by ELISA against recombinant tetNA (y-axis) in pooled sera (x-axis). The titer, averaged from triplicate data, is indicated in the matrix, the blue scale represents normalized values (Titer/highest NA titer against each antigen). (b) HA-specific endpoint IgG titers determined by ELISA against HA components (y-axis) used against pooled sera (x-axis). End point titers are indicated and the blue shade represent the normalized values (Titer/highest NA titer against each antigen). (c) Endpoint IgG subclass titers determined by ELISA against coated homologous recombinant NA or HA immunogen as indicated above each of the graphs. Each data point represents an independent quantification (d). Neuraminidase inhibition determined in ELLA assay using anti-NA sera (x-axis) against the indicated RG IBV strains (y-axis). The values shown are the average IC50 values and the blue shades represent the normalized values (IC50/max IC50 per strain). (e) HI was determined using ether-treated IBV strains (y-axis) and anti-HA sera (x-axis). The values represent endpoint titers and the blue scale the normalized values (HI Titer/ max HI titer per strain).

Serum IgG ELISA titer against immobilized tetNAs were the highest against homologous NAs (Fig 1a). Anti-NA Bris and anti-NA Col sera, both Victoria lineage, showed some level of intra-lineage cross-reactivity and limited cross-reactivity against NA from the Yamagata lineage Phu (Fig 1a). Likewise, the sera raised against Phu tetNA displayed a high homologous titer, and lower, yet detectable, cross-reactivity against tetNAs from the Victoria lineage viruses Col and Bris (Fig 1a).

In parallel, anti-HA immune sera were generated by immunizing mice with 1 μg of adjuvanted rHA in a prime-boost regimen. These HA immune sera were highly reactive in ELISA against the homologous antigens (Fig 1b). Serum raised against the Victoria strains Bris and Col showed high intra-lineage titers against Bris and Col rHA and low cross-reactivity against the Yamagata strain Phu. Conversely, the Phu anti-HA sera reacted poorly against the antigens from the Victoria lineage Bris and Col HAs (Fig 1b). The subclass composition of the sera was determined using homologous antigens in ELISA titration. IgG1 was the predominant immunoglobulin class, followed by IgG2a and IgG3, whereas IgG2b titers against the recombinant HA and NA antigens remained undetectable (Fig 1c).

Anti-NA protection has been associated with the presence of NA-inhibiting antibodies [25]. Therefore, using a panel of 6 distinct reverse genetics (RG) IBV strains that carried HA and NA of the indicated viruses on the genetic backbone of B/Memphis/12/1997, we determined the NA inhibition activity in the anti-NA sera in an enzyme-linked lectin assay (ELLA). The NA activity of the Victoria lineage viruses RG B/Malaysia/2506/2004 (Mal) and B/Brisbane/60/2008 (Bris) viruses was strongly inhibited by sera raised against tetNA Bris. NA of the more recent Victoria lineage RG B/Colorado/06/2017 was strongly inhibited by homologous sera and less by anti-NA Bris and Phu sera. NA activity of the 3 Yamagata representative strains RG B/Florida/4/2006, RG B/Massachusetts/02/2012, and RG B/Phuket/3073/2013 was strongly inhibited by anti-NA Phuket serum and poorly by sera raised against tetNAs from the two Victoria lineage viruses (Bris and Col) (Fig 1d).

The gold standard correlate of protection against influenza is defined by hemagglutination inhibition (HI) titers. We found that homologous serum HI titers were typically higher than heterologous ones (Fig 1e). The pattern of cross-HI observed in sera raised against rHAs Bris, Col and Phu followed a similar pattern to the one observed in the ELLA assay. Hemagglutination activity of the three Victoria lineage viruses RG B/Malaysia/2506/2004, RG B/Brisbane/60/2008, and RG B/Colorado/06/2017 was strongly inhibited by Bris anti-HA sera (Fig 1e). Likewise, the Col HA immune serum exerted clear HI activity against the 3 Victoria lineage RG viruses. The Col HA and Bris HA immune serum have HI activity also observed against the Yamagata lineage RG virus B/Florida/4/2006 (Fig 1e). Conversely, HI titers of the Phu HA immune serum was highest against the Yamagata lineage RG viruses B/Florida/4/2006, B/Massachusetts/02/2012, and B/Phuket/3037/2013 and lowest against the Victoria lineage viruses RG B/Malaysia/2506/2004 and B/Brisbane/60/2008 (Fig 1e). The Phu HA immune serum also displayed relatively high HI activity against the heterologous RG B/Colorado/06/2017 virus (Fig 1e).

In summary, intra-lineage titers in recombinant IBV NA and HA immune sera as determined by ELISA, ELLA, and HI in general were higher than inter-lineage titers.

### Homologous anti-HA and anti-NA serum transfer protects against RG B/Colorado/02/2017 and RG B/Phuket/3073/2013 challenges

**Fig 2:**
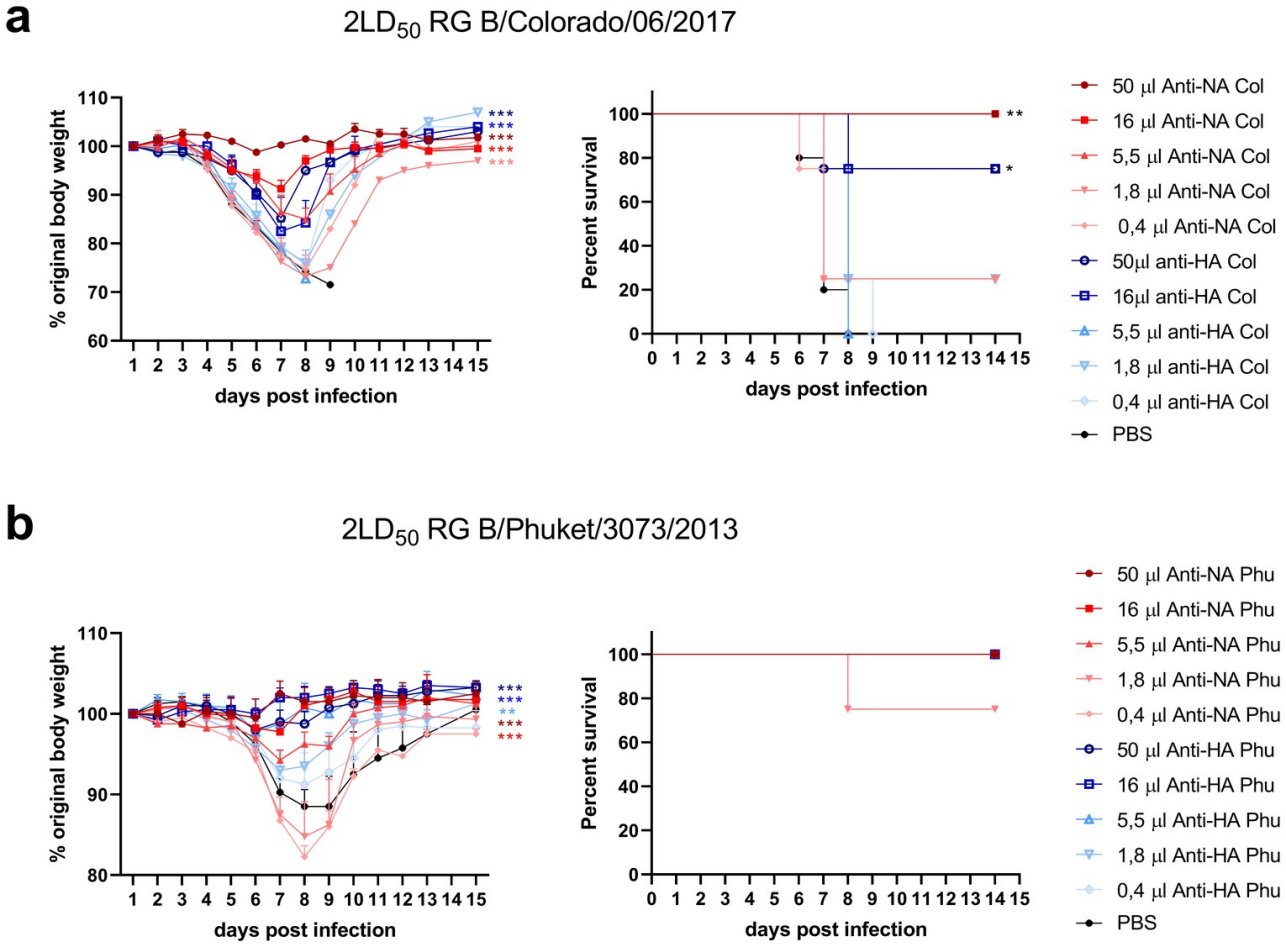
The minimal volume of serum required to confer protection in mice against homologous challenge. BALB/c mice received an intraperitoneal injection of different volumes of anti-NA or anti-HA immune serum ranging from 0.4 to 50μl one day prior to infection with 2LD_50_ of RG (a) B/Colorado/06/2017 or (b) B/Phuket/3073/2013. Relative body weight values were analysed as repeated measurements using the method of residual maximum likelihood (REML), compared to the PBS control group. Survival analysis was done by the log-rank (Mantel-Cox) test. Significances: *P<0.05; **P<0.01; ***P<0.001. Four BALB/c mice were used per group. Data points in the body weight graphs represent averages of the relative percentages of body weight of all mice alive at the time of body weight measurement. Error bars represent s.e.m.

First, we determined the protection provided by different amounts of anti-NA and anti-HA immune sera against homologous challenges. The aim of the titration was to determine the minimal range of serum able to provide protection. This serum dose titration followed by challenge with 2 LD_50_ of RG B/Colorado/06/2017 virus revealed that anti-NA and anti-HA immune sera protected mice against body weight loss in a dose-dependent way, and that a dose of 16μl was sufficient to protect mice from mortality and body weight loss compared to the control PBS group (Fig 2a). Similarly, different amounts of homologous anti-HA or anti-NA sera were transferred to mice one day prior to challenge with RG B/Phuket/3073/2013 virus and were shown to be able to dose-dependently reduce morbidity (Fig 2b). We decided to use a volume of 16 μl of anti-HA and -NA immune serum because this dose provided suboptimal protection against body weight loss, thereby leaving a window that could reveal enhanced as well as reduced protection against intra- and inter-lineage challenges.

### Yamagata anti-NA immune serum can protect against challenge with Victoria lineage viruses

**Fig 3:**
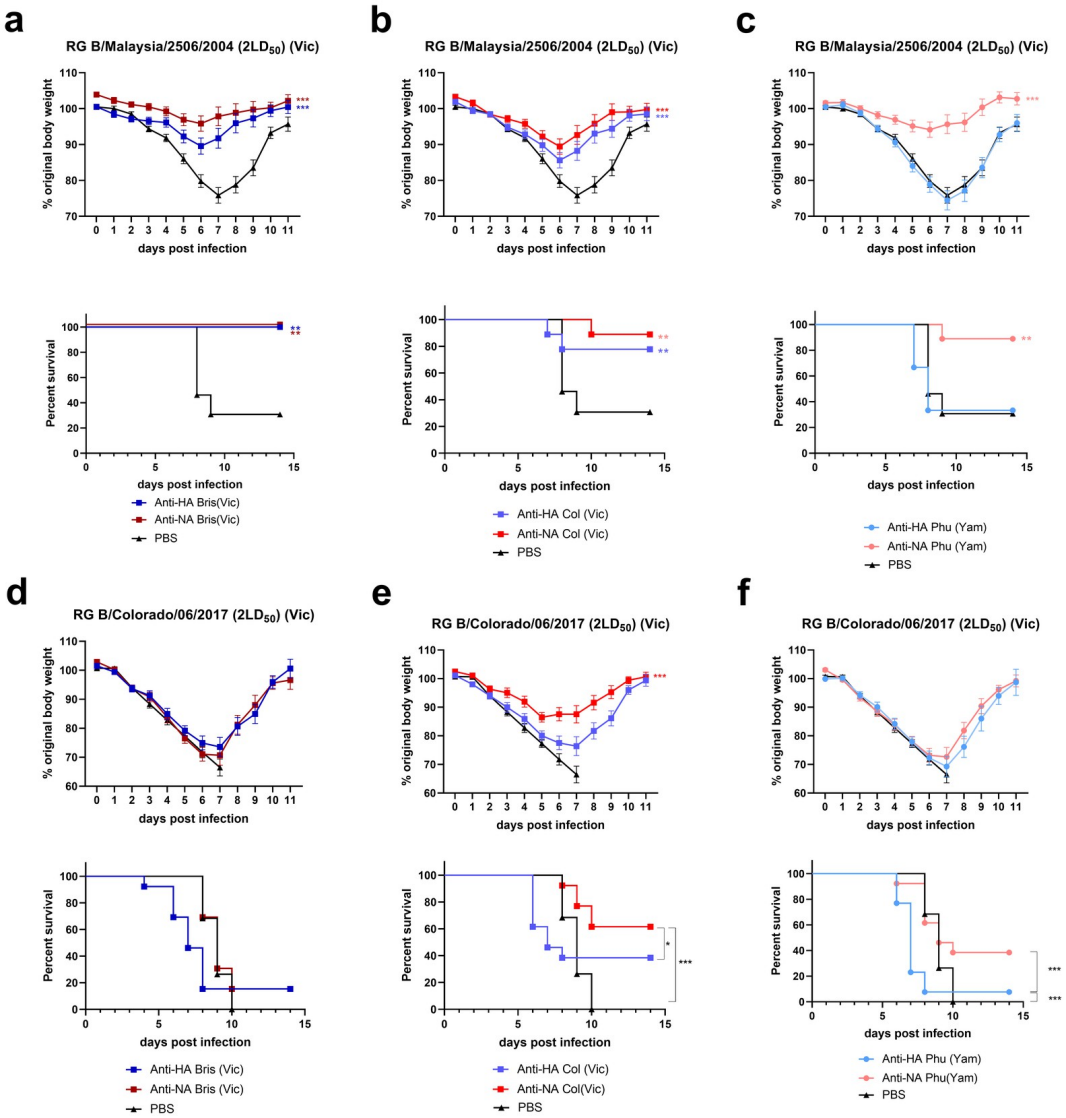
Cross protection induced by serum transfer against representative Victoria lineage viruses. Mice received 16 μl of serum raised against tetNA and HA from B/Brisbane/60/2008 (a and d), B/Colorado/06/2017 (b and e), or B/Phuket/3073/2013(c and f). One day after intraperitoneal injection of the sera, the mice were challenged intranasally with 2LD_50_ of RG B/Malaysia/2506/2004, and weight loss and mortality were determined, experiment was repeated twice with 4–5 mice per group (a-c). Morbidity and mortality were also determined in mice challenged with RG B/Colorado/06/2017. The experiment was repeated 3 times with 4–5 mice per group (d-f). Relative body weight values were analyzed as repeated measurements using method of residual maximum likelihood (REML). Survival analysis was done by the log-rank (Mantel-Cox) test. Significances: **P<0.01; ***P<0.001. The PBS group refers to data pooled from mice that received PBS or serum from naïve mice prior to challenge. All groups receiving serum transfer were tested simultaneously against each challenge, but for clarity the weight loss and survival curves were split in distinct graphs. As a consequence, the PBS control data is repeated.

We next explored the extent and broadness of protection against homologous and heterologous IBV challenge by passively transferred anti-NA and anti-HA immune sera in a mouse model. To evaluate the impact of cross-reactive sera on IBV infection, mice received 16 μl of serum directed against NA or rHA derived from B/Brisbane/60/2008, B/Colorado/06/2017, or B/Phuket/3073/2013, delivered intraperitoneally one day prior to challenge with 2 Victoria lineage strains: RG B/Malaysia/2506/2004 and RG B/Colorado/06/2017 (Fig 3). Anti-HA Bris and Col immune sera protected mice significantly better than PBS treated mice against challenge with RG B/Malaysia/2506/2004 virus, whereas recipients of anti-HA Phu serum were not protected.

Both Victoria (Bris and Col) and, remarkably, Yamagata (Phu) anti-NA sera transfer protected mice against RG B/Malaysia/2506/2004 challenge (Fig 3a–3c). Weight loss was significantly lower in the group of mice that received anti-NA Bris when compared to the groups that received anti-HA Bris sera (p = 0.029) (Fig 3a). The weight loss and survival were also significantly different for the group that received Yamagata anti-NA Phu serum when compared to anti-HA Phu serum (p<0.001) (Fig 3c). The finding that anti-NA Phu serum protected statistically significantly better than anti-HA Phu serum against challenge with RG B/Malaysia/2506/2004 lineage is in line with the ratios of the HI and NI titers against homologous virus over the titers against the heterologous challenge virus. These ratios are 60-fold for HI and 8-fold for NI, respectively (Fig 1d and 1e).

Mice that were challenged with the more recent Victoria lineage RG B/Colorado/06/2017 virus had significantly reduced weight loss and mortality when compared to the PBS group after receiving homologous Col anti-NA serum whereas mice that had received homologous Col anti-HA immune serum were not protected (Fig 3e). Mice challenged with RG B/Colorado/06/2017 that received anti-Phu or anti-Bris sera (anti-HA or NA) were not protected (Fig 3d and 3f). However, the groups that received heterologous anti-NA serum had increased survival when compared to mice that received the corresponding anti-HA serum (Fig 3d and 3f, p = 0.046 and p = 0.0012 for Bris and Phu sera respectively). The difference observed in anti-HA versus anti-NA Bris serum transfer reflect the early onset of morbidity in mice that received anti-HA Bris sera.

Overall, transfer of Victoria lineage anti-NA serum resulted in slightly better protection than anti-HA serum against challenge with the Victoria lineage RG B/Malaysia/2506/2004 and RG B/Colorado/06/2017 virus, but this difference was not statistically significant (Table 1).

**Table 1:** Survival of mice that received anti-HA or -NA serum after challenge with RG Yamagata or Victoria viruses.

sera		Challenge strains						
		Col17	Mal04	Phu13	Mas12	Flo06	All Vic	All Yam
Bris08	HA	2/13 (15.4%)	8/8 (**100%**)	4/9 (44.4%)	2/9 (22.2%)	3/8 (37.5%)	13/26 (**50%**)	9/26 (34.6%)
	NA	2/13 (15.4%)	9/9 (**100%**)	8/9 (**88.9%)**	2/9 (22.2%)	6/8 (**75%**)	16/27 (**59.3%**)	16/26 (**61.5%**)
Col17	HA	5/13 (38.5%)	7/9 (**77.8%**)	3/9 (33.3%)	1/9 (12.1%)	3/9 (37.5%)	16/27 (**59.3**%)	7/26 (26.9%)
	NA	8/13 (**61.5%**)	8/9 (**88.9%**)	6/9 (**66.7%**)	2/7 (22.2%)	0/8 (0%)	21/27 (**77.8**%)	8/26 (30.8%)
Phu13	HA	1/13 (7.7%)	3/9 (33.3%)	9/9 (**100%**)	9/9 (**100%**)	8/8 (**100%**)	4/27 (14.8%)	26/26 (**100**%)
	NA	5/8 (38.5%)	8/9 (**88.9%**)	8/9 (**88.9%**)	8/9 (**88.9%**)	8/8 (**100%**)	15/27 (**55.5%**)	20/26 (**76.9%**)

### Protection conferred by serum transfer against challenge with Yamagata lineage viruses

To evaluate the potential cross protection induced by serum transfer against yamagata challenges, mice received immune serum raised against Bris, Col, or Phu HA or NA prior to challenge with RG B/Florida/04/2006, RG B/Massachusetts/02/2012, or RG B/Phuket/3073/2013 RG strains.

**Fig 4:**
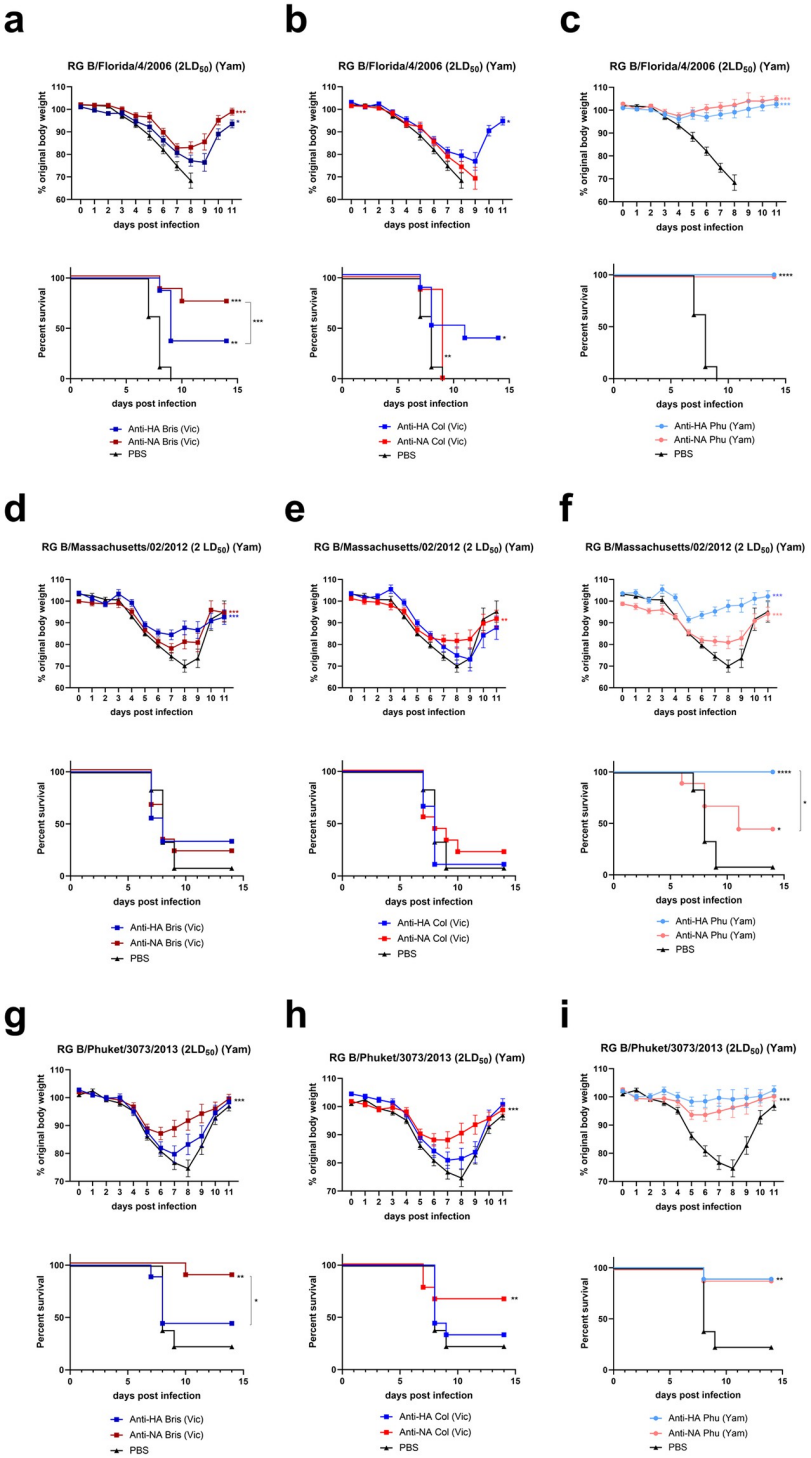
Cross protection provided by serum transfer against representative Yamagata lineage viruses. Mice were injected intraperitoneally with 16 μl of serum raised against tetNA or HA from B/Brisbane/60/2008 (a, d and g), B/Colorado/06/2017 (b, e and h), or B/Phuket/3073/2013(c, f and i). One day after intraperitoneal injection of serum, the mice were challenged intranasally with 2LD_50_ of RG B/Florida/4/2006 (a-c), BALB/c mice), RG B/Massachusetts/02/2012 (d-f, DBA2/J mice), or RG B/Phuket/3073/2013 (g-i, BALB/c mice). Relative body weight values were analyzed as repeated measurements using method of residual maximum likelihood (REML). Survival analysis was done by the log-rank (Mantel-Cox) test. Data represent Significances: *P<0.05; **P<0.01; ***P<0.001; ****P<0.0001). Each challenge experiment was repeated twice with 4–5 mice per group. The PBS group refers to data pooled from mice that received PBS or serum from naïve mice prior to challenge. All groups receiving serum transfer were tested simultaneously against each challenge, but for clarity the weight loss and survival curves were split in distinct graphs. As a consequence the PBS control data is repeated.

The mice that received Bris anti-NA, Bris anti-HA, or Col anti-HA immune serum were partially protected against challenge with RG B/Florida/04/2006 based on survival outcome, despite substantial weight loss (Fig 4a and 4b). The mice that had received serum directed against Phu NA or HA before challenge with RG B/Florida/04/2006 were fully protected against weight loss and mortality (Fig 4c). When comparing survival HA versus NA serum pairs, only the groups that received anti-Bris are statistically significantly different from each other (p = 0.001, anti-HA vs NA survival) with anti-NA being more protective (Fig 4a).

RG B/Massachusetts/02/2012 was not pathogenic in BALB/c mice and for that reason the DBA/2J mouse strain, which is more susceptible to influenza virus challenge, was used to assess protection by the anti-NA and anti-HA immune sera [26]. Weight loss was significantly reduced in the groups that received heterologous anti-HA or NA Bris serum when compared to the PBS group but no difference was observed in survival (Fig 4d). The group that received Col anti-NA serum had a slightly reduced morbidity compared to the PBS group, while the group that received Col anti-HA serum behaved similarly to the PBS group (Fig 4e). Phu anti-HA serum transfer protected mice from weight loss and mortality, while Phu anti-NA serum conferred only partial protection against weight loss and mortality following RG B/Massachusetts/02/2012 challenge (Fig 4f). Protection conferred by anti-HA Phu sera was superior to the protection induced by anti-NA Phu sera (p<0.001 and p = 0.01, for weight loss and mortality, respectively).

We also assessed protection by the anti-HA and anti-NA immune sera against challenge with RG B/Phuket/3073/2013 virus. The weight loss of all the groups that received serum differs from the PBS group and this protection is maximal for the groups that received homologous Phu anti-NA or anti-HA serum (Fig 4g–4i). Body weight loss was significantly lower between mice that had received Bris anti-NA serum compared with Bris anti-HA serum (Fig 4g, p = 0.033) and between mice that had received Col anti-NA serum compared with Col anti-HA serum (Fig 4h, p<0.001). Statistically significant protection against mortality was observed for the groups that received homologous Phu anti-NA and anti-HA serum and for the mice that received Bris or Col anti-NA serum (Fig 4g–4i).

### Infection of primary human airway epithelial cells is restricted by homologous anti-NA and anti-HA immune sera

**Fig 5:**
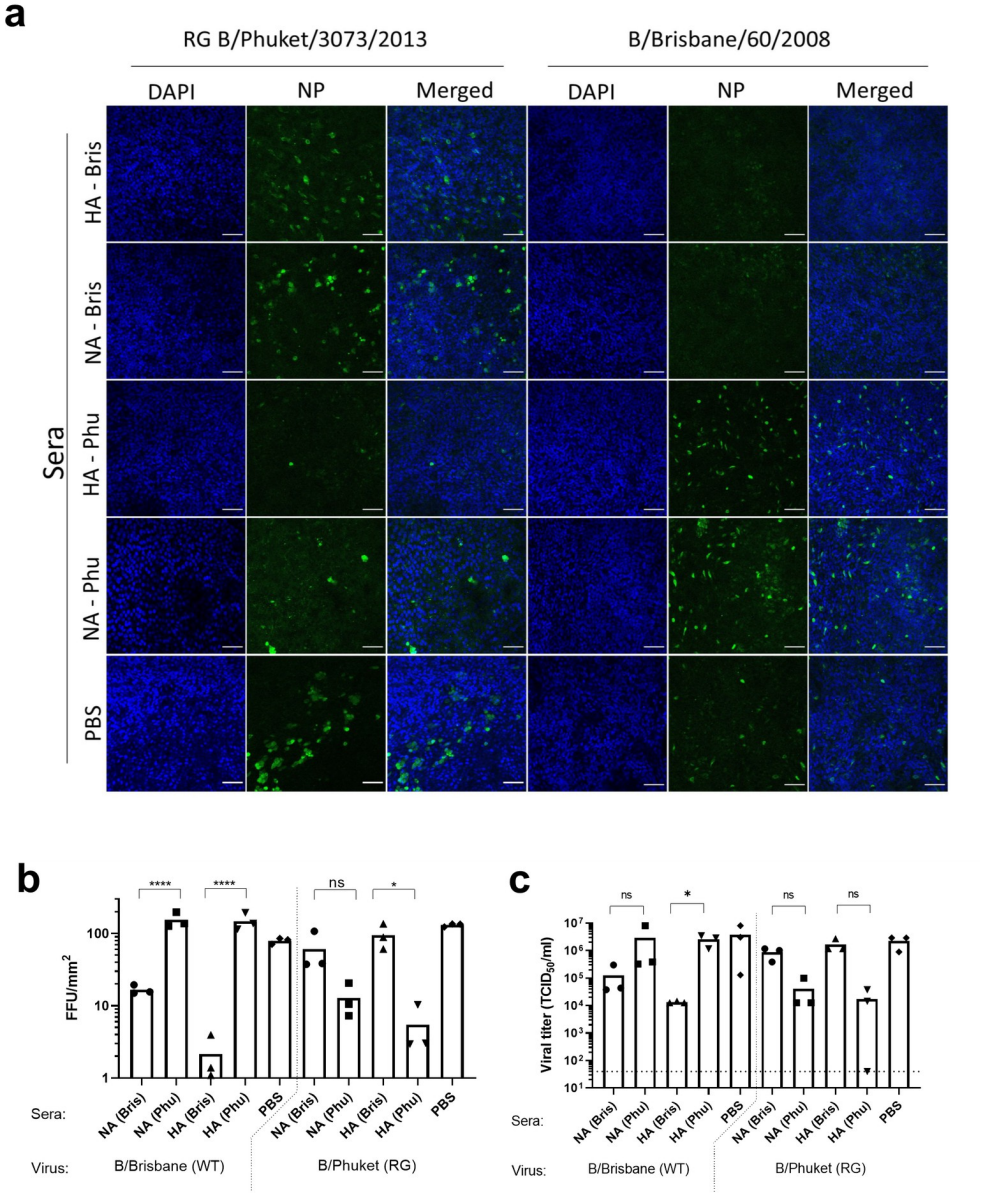
Homologous anti-HA and anti-NA immune serum restricts IBV infection of HAE cells. HAE cells were infected with RG B/Phuket/3073/2013 or B/Brisbane/60/2008 in the presence of the indicated anti-NA, anti-HA or PBS mouse serum. (a) Infection was imaged by NP staining. (b) The number of focus-forming units (FFU) were quantified using QuPath 0.3.0 software. (c) Titers of virus present in washes of the apical side of the HAE cells taken at 24 after infection. *P<0.05 and ****P<0.0001 (one-way ANOVA).

It has been reported that antibodies directed against HA and NA can restrict influenza A virus infection in primary human airway epithelial cells [27, 28]. To assess whether influenza B virus infection can also be hindered by HA- and NA-specific antibodies, we infected HAE cells with 0.1 MOI of RG B/Brisbane/60/2008 or RG B/Phuket/3073/2013 viruses in the presence or absence of a 1:200 dilution of receptor destroying enzyme-treated mouse serum raised against homologous or heterologous IBV HA or NA. Twenty four hours after infection, samples were taken from the apical side on the HAE cultures for virus titration and the cells were fixed and immune-stained for the presence of IBV nucleoprotein positive foci. Quantification of IBV focus forming units after nucleoprotein staining shows that Bris anti-NA and anti-HA sera significantly reduced infection of RG B/Brisbane/60/2008 (Victoria) virus when compared to the Phu anti-NA and anti-HA sera or sera from mock immunized mice (PBS) (Fig 5). Conversely, homologous Phu anti-HA serum could significantly reduce infection of RG B/Phuket/3073/2013, but heterologous Bris anti-HA serum failed to do so (Fig 5b). A similar trend was observed in HAE cells treated with homologous Phu anti-NA serum, but statistical significance was not reached in the assay (Fig 5b). Homologous anti-HA immune serum could reduce the production of virus sampled from the apical side of the HAE cultures although statistically significant differences compared with the PBS serum treated samples were not reached (Fig 5c).

## Discussion

Phylogenetic analysis has revealed that Victoria and Yamagata IBV lineages evolve along different pathways. While Victoria lineage viruses evolves by drastic changes in HA and NA, the Yamagata strains experience faster seasonal fluctuations, especially in the NA protein [12].

Our serological analysis revealed a limited inter-lineage cross-reactivity for both HA and NA components. This indicates that, despite the incorporation of NA from Yamagata by the Victoria lineage viruses in the early 2000s, the NA has antigenically diverged considerably in the tested strains.

Despite the limited inter-lineage cross-reactivity, the serum raised against Bris and Phu HA or NA showed intra-lineage cross-inhibition which was less pronounced in the sera raised against the most recent B/Colorado/02/2017 HA and NA. This observation agrees with the notion that more recent IBV Victoria lineage strains follow an independent divergent evolutionary trajectory, characterized by HA deletions and mutations which are accompanied by compensatory mutations in NA [12]. Cross-inhibition titers of anti-HA and NA sera follow a similar pattern, as an evidence of co-evolution.

In terms of cross-protection, a volume-based amount of sera was transferred to mice prior challenges. This fact has not biased the results as consequence of the homologous titer. Furthermore, the volume-based amount of sera used in the transfer experiments also reflects the intrinsic immunogenicity of each antigen, a characteristic that cannot be modulated in field trials. The serum transfer showed that the Bris anti-NA sera tended to induce a broader protection against the heterologous Yamagata strains when compared to Bris anti-HA sera. Similarly, Phu anti-NA tended to induce some level of cross-protection against Victoria strains, unlike Phu anti-HA sera. This observation suggests that despite the low level of cross-reactive antibodies, some level of cross-protection is induced by inter-lineage humoral NA immunity. We also note that Phu anti-HA serum protected significantly better than Phu anti-NA serum against intra-lineage challenge with RG B/Massachusetts/02/2012 (Fig 4d–4f). This result was obtained in DBA/2J mice, which, unlike BALB/c mice, developed disease with this challenge virus, even though a very high challenge dose of 1.3 x 10^6^ PFU/mouse was required. It should be noted that the intrinsic nature of each challenge strain limits the inter-challenge comparisons. Negative control groups received PBS or serum from naïve mice. No protection against subsequent IBV challenge was observed in these two groups, excluding potential unspecific effects of serum components. In all challenge models the observed cross-protection was not strong enough, by both HA and NA sera, to completely inhibit weight loss, indicating that infection-associated pathology still occurs.

This is in line with the results obtained *in vitro* using HAE cells as a model. In that case we could observe that anti-HA or anti-NA sera could restrict infection by homologous strains but not by a heterologous strain. Despite the similar composition of Ig subclasses among the anti-HA or NA immune sera, further investigation is required to determine the mechanisms involved in cross-protection observed *in vivo*, which may depend on Fc-mediated effector functions [29].

In summary, optimal protection is achieved with a homologous serum transfer and little inter-lineage cross-protection is observed in the majority of the performed challenges. Another important conclusion is that the anti-NA immune serum was at least as performant as anti-HA immune serum in controlling IBV-induced morbidity and lethality. In addition, anti-NA sera performed better than anti-HA sera to prevent mortality in inter-lineage heterologous challenges with 4 out of 5 distinct IBV reassortants. The protective effect of anti-NA serum transfer confirmed the importance of anti-IBV NA immunity. In line with those observations, the increased nucleotide substitution rate on IBV NA, when compared to HA, suggests that NA is under stronger antigenic pressure and stresses the significant role of NA on IBV drift [12].

The recent divergence of the Victoria lineage HA into distinct clades and the faster evolution of recent Yamagata strains complicate decisions on influenza vaccine composition. In addition, the recent sanitary restrictions imposed due to the COVID-19 pandemic may impact the evolutionary trajectories of IBV, potentially leading to extinction of the IBV Yamagata lineage strains and opening space for unpredicted evolutions [30]. The incorporation of NA as a vaccine antigen may, in the future, compensate for possible mismatches and improve the breadth of protection, maximizing protective effectiveness and its benefits that goes beyond the flu [6, 24, 31].

## Materials and methods

### Viruses

The HA and NA segments of B/Florida/4/2006, B/Massachusetts/02/2012, B/Phuket/3073/2013, B/Malaysia/2506/2004, B/Brisbane/60/2008, and B/Colorado/06/2017 were synthetically constructed and inserted in the bi-directional expression vector pHW2000 [32]. A co-culture of MDCK and HEK293 cells was then transfected using 1 μg of each HA, NA and the plasmids coding for the remaining segments of mouse adapted B/Memphis/12/1997 [33]. Supernatant was recovered after observation of cytopathic effect and the rescued viruses were amplified on MDCK cells to prepare working stocks. Briefly, MDCK cells were inoculated with a MOI of 0.001 in the presence of TPCK-treated trypsin (2μg/ml, Sigma T1426-50MG). After cytopathic effect was evident, the cell culture supernatant was recovered and cleared from cells and debris by centrifugation at 1240 RFCs. The cleared supernatant was then centrifuged at 18380 RFCs for 90 minutes and the pelleted virions resuspended with 7% Glycerol in PBS. All HA and NA sequences in the obtained RG viruses were confirmed by Sanger sequencing.

### Recombinant proteins

Recombinant HA monovalent component derived from B/Phuket/3073, B/Brisbane/60/2008, and B/Maryland/15/2016 (a B/Colorado/06/2017-like strain referred in the main text as “Col”) were produced by Sanofi. These were the same drug substances that are being used in the Flublok drug product sold by Sanofi.

The recombinant NAs were expressed as stalk-free enzymatic head domains (amino acids 70 to 465) that were stabilized by fusion to the tetrabrachion domain [34, 35]. The coding sequence for the tetNA proteins were cloned under the transcriptional control of the CMV promoter in the pCDNA3.4 plasmid with a CD5 secretion signal, an amino-terminal His-tag followed by a thrombin cleavage signal. TetNA derived from B/Phuket/3073, B/Brisbane/60/2008, and B/Colorado/06/2017 were expressed in a mammalian cell culture system as previously described with modifications [36].

### Mouse immunization and challenges

All animal experiments were conducted according to the Belgian legislation (Belgian Law 14/08/1986 and Belgium Royal Decree 06/04/2010) and European legislation on protection of animals used for scientific purposes (EU directives 2010/63/EU and 86/609/EEC). Experimental protocols were all approved by the Ethics Committee of the Vlaams Instituut voor Biotechnologie (VIB), Ghent University, Faculty of Science (permit number EC2020-061). Female BALB/c mice, aged 6–7 weeks, were purchased from Charles River (France) and female DBA/2J mice aged 6–7 weeks were purchased from Janvier (France).

To generate immune sera, 20 BALB/c mice per group were intramuscularly immunized in a prime-boost regimen with 1 μg of AF03 adjuvanted tetNA or rHA protein with a 2 weeks interval. Mice were terminally bled 2 weeks after the second immunization.

The LD_50_ dose of the RG IBVs was determined by infecting 6–8 weeks old female BALB/c or DBA/2J mice with different doses of viruses in a 3 fold serial dilution. The ethical endpoint for euthanasia of the mice was reached when mice had lost 25% or more of their body weight relative to day of challenge. Mice were humanely euthanized by cervical dislocation. The amount of virus corresponding to 1LD50_50_ is 5.1 x 10^4^ for B/Colorado/6/2017 (BALB/c), 1.6 x 10^4^ for B/Malaysia/2506/2004 (BALB/c), 3.5 x 10^3^ for B/Florida/4/2006 (BALB/c), 1.3 x 10^6^ for B/Massachusetts/02/2012 (DBA/2J), and 8.6 x 10^4^ for B/Phuket/3073/2013 (BALB/c).

For the challenge experiment, the mice received serum diluted in PBS in a total volume of 100μl by intraperitoneal injection one day prior to infection. In the first set of experiments control mice received PBS whereas in the repeat experiment(s) the control group received serum from naive mice. The results of mice that had been injected with PBS or naïve serum were pooled and referred in the main text as “PBS” groups. For virus inoculation, the mice were anesthetized with 5% isoflurane and 50μl of virus dilution was applied into both nostrils ensuring complete aspiration. Body weight change was determined daily for 14 days after infection. Mice were euthanized when they had lost more than 25% of body weight or were sacrificed 14 days after infection.

#### Repeated measurements and survival analysis of challenge data

Each challenge experiment was repeated two or three times (as referred in the figures lengeds), with 4–5 mice per challenge and per replicate. Relative body weight values were analyzed as repeated measurements using method of residual maximum likelihood (REML), as implemented in Genstat for Windows 21^st^ edition. Briefly, a linear mixed model of the form y = replicate + challenge + time + challenge × time + mouse × time was fitted to the repeated measurements. The term mouse × time represents the residual error term with dependent errors because the repeated measurements are taken in the same mouse, causing correlations among observations. Several covariance models were fitted to the data to account for the correlation present in the data. The antedependence correlation model of order 2 (ANTE2) was finally selected as best fitted model based on the Akaike’s information criterion coefficient. The ANTE covariance model assumes that correlation between observations decays as the measurements are collected further apart in time, just like the autoregressive covariance model does, but allows for changes in correlation structure over time. The second order refers to the fact that recent (*t*-1) and less recent (*t*-2) observations affect the current observations made at timepoint *t*. The significance of the fixed terms in the model and significance of changes in difference between challenge effects over time, were assessed using an approximate *F*-test as implemented in Genstat for Windows 21^st^ edition. Differences in survival was detenimed using Mantel-Cox log rank test perfomed in Graphpad version 8.00 for Windows (GraphPad Software, San Diego California; www.graphpad.com).

### ELISA

Anti-tetNA and anti-HA IgG levels in mouse sera were determined by ELISA as follows. Recombinant tetNA was diluted in DPBS (Life technologies cat. # 14040–182) to 0.5μg/ml and 50μl was added to each well of a MaxisorpTM plate (ThermoFisher cat. # 44-2404-21). The plates were incubated at room temperature for 1h on a shaking platform. Recombinant HA was diluted in coating buffer (KPL cat # 50-84-00) to 0.5μg/ml and 50μl was added to each well of a MaxisorpTM plate (ThermoFisher cat. # 44-2404-21). After an overnight incubation at 4°C, the wells of the plates were washed three times with PBS-T (Sigma cat. # P3563-10PAK) and blocked for 1 h with 1% BSA in DPBS. After blocking, the wells of the plates were washed once with PBS-T and incubated with a threefold serial dilution, starting at a 1/100 dilution, of serum in DPBS, 0.5% BSA, 0.05% Tween 20 for 2 h at room temperature on a shaking platform. The wells of the plates were then washed five times with PBS-T and incubated with a 1:5000 dilution of anti-mouse IgG-HRP (GE healthcare cat. # NA931-1ml), anti-mouse IgG3-HRP (SouthernBiotech cat. # 1100–08), anti-mouse IgG2a-HRP (SouthernBiotech cat. #1080–05), anti-mouse IgG2b-HRP (SouthernBiotech cat. # 1090–05), anti-mouse IgG1-HRP (SouthernBiotech cat.#1070–01) in PBS, 0.5% BSA, 0.05% Tween20. The 3,3′,5,5′-tetramethylbenzidine substrate (TMB) (BD cat. # 555214) was added after three washes with PBS-T and the reaction was stopped after 5 min by addition of 50 μl of 1 M H_2_SO_4_. The optical density (OD) in each well was determined at 450 nm and, as a reference, 655 nm using an iMark^™^ Microplate Absorbance Reader (Bio-rad). The end point titer was determined for each serum sample by scoring the dilution that resulted in an OD that was equal to or higher than two times the background OD obtained from the pre-immune control serum dilution series.

### Enzyme-linked lectin assay to determine neuraminidase inhibition titers

Fetuin (Sigma cat. # F3385) was diluted into coating buffer (KPL cat. # 50-84-01) to a concentration of 25 μg/ml and 50 μl was added to the wells of Nunc MaxiSorp^™^ plates (Thermofisher cat. # 44-2404-21), which were incubated overnight at 4 °C. The coated plates were then washed three times with PBS-T (Sigma cat. # P3563-10PAK) and incubated overnight with 25 μl of virus dilution corresponding to 70% maximal sialidase activity and 25 μl of 2-fold serial dilution of serum, starting at a 1/20 dilution, in sample buffer (1X MES VWR cat. # AAJ61979-AP: 20 mM CaCl_2_, 1% BSA, 0.5% Tween 20, pH 6.4). Fetuin-coated plates were then washed three times with PBS-T and incubated for 1 h with a solution of PNA-HRP (cat. # L6135-1MG, Sigma) at 5 μg/ml in conjugate diluent (MES pH 6.5, 20 mM CaCl2, 1% BSA). The plates were washed three times with PBS-T, TMB substrate was added after which the plates were incubated for 5 min before the reaction was stopped by the addition of 50 μl of 1 M H_2_SO_4_. The optical density was measured at 450 nm and, as a reference, 655 nm in an iMark^™^ Microplate Absorbance Reader (Bio-rad). Half maximum inhibitory concentration (IC_50_) values were determined by nonlinear regression analysis using GraphPad Prism software.

### Hemagglutination inhibition assay

Hemagglutination and HI tests were performed in a round bottom 96-well microtiter plate at room temperature using 1% (vol/vol) turkey erythrocytes in PBS with 4 hemagglutinating units (HAU) of ether-treated RG IBV viruses according to the WHO manual for influenza research [37].

### HAE cells infection

Differentiated HAE cells (Mucilair) originated from healthy donors were purchased from Epithelix. The cells were incubated at 37°C with 5% CO_2_ in MucilAir culture medium (Epithelix), with media refreshment in the basal chamber every 2–3 days. The cells were infected with a MOI of 0.1 (5x10^5^ PFUs) during 24h in the presence of anti-NA, anti-HA or PBS-immunized mouse sera. The apical side of the HAE cells was washed with PBS 24h after infection and the resulting SN was titrated in MDCK cells.

HAE cells were fixed in 4% PFA for 1h, permeabilized with 0.15% Triton X100 and stained for imaging by fluorescent microscopy using anti-IBV NP (MA1-80712, ThermoScientific, 1:1000 dilution). Hoechst co-stain was used to visualize the nucleus. HAE support membranes were finally cut from their supports and mounted on glass slides for microscopy. Membranes were imaged using Zeiss AxioScan slide scanning and FFU were quantified with QuPath 0.3.0 software. Illustrative pictures were randomly acquired using a Zeiss LSM880 Fast Airy Scan confocal microscope.
